# Controlling the Nanoscale Gaps on Silver Island Film for Efficient Surface-Enhanced Raman Spectroscopy

**DOI:** 10.3390/nano9030470

**Published:** 2019-03-20

**Authors:** Yu-Chung Chang, Yu-Chun Lu, Yu-Ju Hung

**Affiliations:** 1 Department of Electrical Engineering, National Changhua University of Education, Changhua 500, Taiwan; 2 Department of Photonics, National Sun Yat-sen University, Kaohsiung 804, Taiwan; sm11240@hotmail.com

**Keywords:** surface-enhanced Raman scattering (SERS), silver island film, surface plasmon resonance

## Abstract

We control the nanoscale gaps on silver island films by different processing methods and investigate the surface-enhanced Raman scattering (SERS) efficiency on the films. We propose a facile technique to control the film morphology by substrate bending while keeping the evaporation rate constant. The films developed by our new method are compared to the films developed by traditional methods at various evaporation rates. The SERS signals generated on the samples prepared by the new method have similar strengths as the traditional methods. Substrate bending allows us to reduce the gap sizes while using a higher evaporation rate, hence the film can be developed in a shorter time. This cost-effective and time-efficient method is suitable for the mass production of large-area SERS sensors with good sensitivity. Scanning electron microscope images are analyzed to quantify the gap densities and widths to elucidate the relationship between the film morphology and the SERS intensity. While the gap size appears to be the major factor influencing the enhancement, the shape of the nano-island also seems to influence the SERS efficiency.

## 1. Introduction

Surface-enhanced Raman scattering (SERS) spectroscopy is a powerful molecular analytic tool which has shown great application potentials in chemistry, physical and biological sciences, environmental monitoring, and medical diagnostics, etc. Since the extraordinary single molecule detection sensitivity was reported by Nie and Emory and Kneipp et al. using SERS [1,2], we have witnessed an explosion in research activity in the field of plasmon-enhanced spectroscopy in the last two decades. Related applications and more detailed descriptions about the mechanism can be found in several excellent review articles [3,4,5]. Recently, owing to the unique properties of label-free and high spatial resolution, SERS has been exploited as a tool for non-destructive intracellular live imaging [6]. A lot of effort has been made to design nanostructures with metals (usually Ag, Au, and Cu) to maximize the SERS efficiency. The interaction between the incident light and the metallic nanostructure gives rise to a significant enhancement in the local field on the metal surfaces due to the excitation of surface plasmon resonance (SPR) [7]. By placing the analytes in the nanoscale gap between metallic nanostructures, the SERS signal can be boosted due to plasmonic coupling effect. These nanogaps are called the “hotspots.” The electrical fields at the hotspots are greatly enhanced due to localized surface plasmon resonance (LSPR) [8,9].

The SERS enhancement factor (EF) is extremely sensitive to the size of the nanoscale gap. Recently, there have been intensive studies focusing on generating controllable nano-sized gaps on coupled nanostructures or nano-patterned surfaces [3,10]. The SERS intensities from these nanogaps are typically two to four orders of magnitude larger than those from single nanostructures [3,11]. With advances in nano-fabrication techniques, many novel SERS substrates have been developed. For example, chemical synthesis-based nanoparticle (NP) self-assembly is capable of producing highly ordered NP superlattice with an EF up to 10^9^ [10,12]. E-beam lithography is capable of producing well-controlled nanopatterns with sub-10 nm features, and is thus widely adopted to fabricate SERS-active substrates [13,14]. In addition, due to the extremely high surface area offered by nanoporous gold (NPG) thin film, analytes can easily bind to the nanosized pores. NPG films are widely used in various applications which require high sensitivities and bio-compatibility [15,16]. Although reproducible SERS substrates with high EF could be achieved with these sophisticated techniques, they are limited by low throughput, expensive production cost, and small active area. For commercial applications, it is desirable to have SERS-active substrate, which is facile to fabricate, low-cost, and reproducible with high yield.

Compared to the sophisticated nano-fabrication techniques mentioned above, silver island films produced by thermal evaporation have naturally-formed nanostructures and nanoscale gaps in a large area. The preparation of silver island films is relatively facile and inexpensive, and thus is ideal for mass production. The morphology of the film can be controlled by the processing parameters, such as substrate temperature, deposition rate, and film thickness. There have been a lot of studies focused on the investigation of the influence of the parameters on the efficiency of the SERS effect [17,18,19]. The resonance frequency of the film substrate can also be fine-tuned by controlling the morphology [20,21]. It is possible to design the film according to the requirement in specific applications. Although it is difficult to control the shape of the silver island in a precise manner, because the film formation obeys the Volmer–Weber mechanism [22], consistent film quality can be obtained with well-controlled experimental conditions. Because the signal we observed is an averaged result from a large number of hotspots randomly distributed on the film, the results are quite robust and repeatable.

In this paper, we propose a new method to have additional controls on nanoscale gap sizes and gap density while keeping other parameters unchanged. By bending the glass substrate with various curvatures, we can generate films with different gaps sizes and densities as well as island geometries. This method allows us to generate films with small nanogaps using a higher evaporation rate, hence the deposition duration can be shortened. With optimized deposition parameters, the produced SERS substrates exhibited excellent uniformity and reproducibility with small standard deviation values of about 3%. The films generated with the new method are compared to films generated by traditional method and show great SERS efficiency. Unlike nanoparticles, due to the irregular shape of the islands, it is difficult to quantify the morphology of the films. We develop an algorithm to analyze the gap size and density of the silver island film to clarify the relationship between the film morphology and the SERS signal. It is found that gap size and density were not the only factor affecting the SERS efficiency, the three-dimensional shape of the nano-island and the mechanical strain on it might also influence the SERS effect [23]. This simple method can be readily employed to produce low-cost large-area SERS-active substrates with high throughput and reproducibility for practical applications.

## 2. Materials and Methods

**Figure 1:**
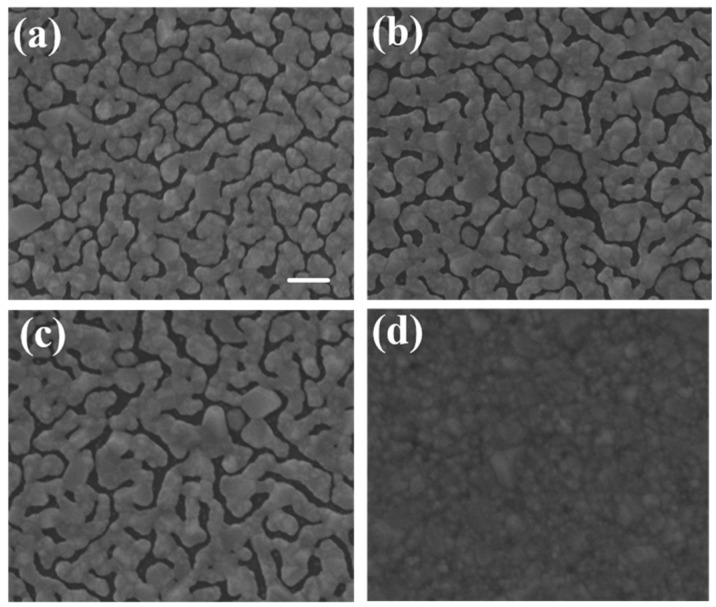
SEM images of sliver film with thickness of 30 nm deposited on flat substrate by thermal evaporation at rates of (**a**) 1.2 Å/s, (**b**) 2.4 Å/s, and (**c**) 3.6 Å/s, labeled as sample A, B, and C, respectively. (**d**) Flat silver film with thickness of 30 nm deposited by E-gun evaporation. Scale bar: 100 nm.

**Figure 2:**
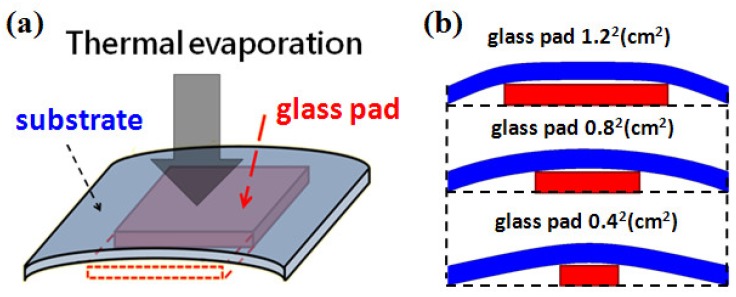
(**a**) Schematic diagram of substrate with glass pad underneath during thermal evaporation. (**b**) Bending geometry with glass pads in different sizes, the maximum curvature occurs around the corners of the underneath glass pad.

We used typical cover glass with a thickness of 150 μm as the substrates. The area was 22 mm × 22 mm. The silver island films were deposited by thermal evaporation. We first developed the silver island film with typical method. The film thickness was fixed as 30 nm and three evaporation rates, 1.2 Å/s, 2.4 Å/s, and 3.6 Å/s, were used in this study. The SEM images of the developed thin films are shown in Figure 1a–c, the samples are labeled as A, B, and C, respectively. We then tried to control the gap density and size by bending the substrate. The substrates were bent in different curvatures during film formation. The evaporation conditions were the same. The bending was caused by simply placing a glass pad under the substrate and fixing two opposite-side edges of the substrate with heat-resistant tapes, as schematically shown in Figure 2a. After removing the tapes, the developed substrate became flat again and ready for spectroscopic measurements.

**Figure 3:**
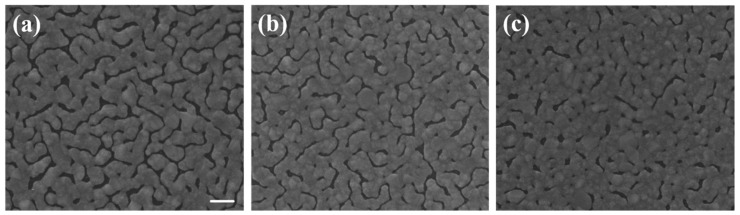
SEM images of sliver films with thickness of 30nm deposited on bent substrates using a fixed thermal evaporation rate at 1.2 Å/s with (**a**) glass pad area 1.2^2^ cm^2^, (**b**) glass pad area 0.8^2^ cm^2^, and (**c**) glass pad area 0.4^2^ cm^2^ underneath, respectively. They are labeled as sample D, E, and F, respectively. Scale bar: 100 nm.

The bending curvature was controlled by the size of the thick glass pad underneath. A smaller glass pad would induce larger bending curvature. The larger the bending curvature, the smaller the gap size and gap density after substrate flattening. Because of the method used for bending, the curvature was not uniform throughout the substrate as shown in Figure 2b. In order to avoid the effects from the non-uniformity, we only measured and took SEM images in the central areas of each sample. Figure 3a–c were the SEM images of island films evaporated with glass pads of areas of 1.2^2^, 0.8^2^, and 0.4^2^ cm^2^ underneath, which are labeled as D, E, and F, respectively. In order to confirm that the spectrum was caused by SERS on sliver nano-islands, we used E-gun evaporator to deposit a flat thin film with the same thickness of 30 nm. The SEM image of the thin film was shown in Figure 1d. Note that the magnification of the SEM images was 2 × 10^5^ times. In this case, the imaging area was about 1.5 μm × 1.5 μm, which was about the size of the laser spot.

Thermal evaporation was carried out with a home-built thermal evaporator. The vacuum pressure for thermal evaporation was 5 × 10^−6^ torr. The current for the heater was 150–175 A depending on the deposition rate. On the other hand, the initial vacuum pressure for the E-gun evaporation was 2 × 10^−6^ torr. The current and voltage of the RF-power was 10 mA and 9 kV, respectively. The E-gun deposition rate was 0.5 Å/s.

**Figure 4:**
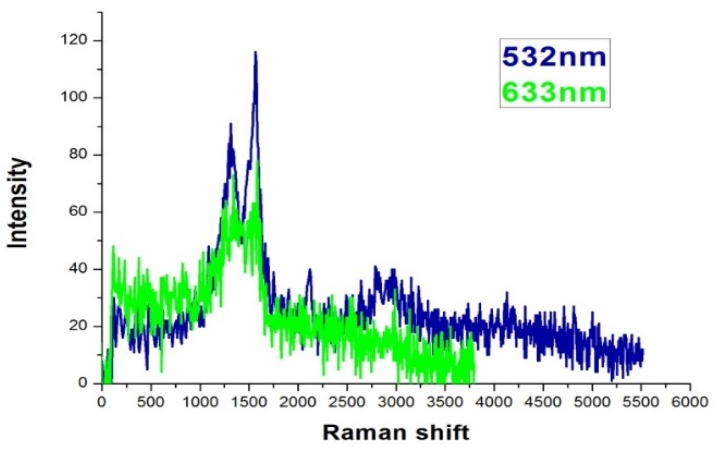
Raman spectra of a silver island film excited by 532 nm and 633 nm CW lasers. The exposure time was 10 s.

The spectroscopic data were taken with a scanning confocal Raman optical microscope (Nanofinder 30, TII Tokyo instruments Inc., Tokyo, Japan). The excitation wavelength of the laser was 532 nm if not specifically noted. Figure 4 is the Raman spectra of as-deposited sample A. The features at 1340 and 1580 cm^−1^ are prominent, which are the D and G band signals from carbon as reported in previous literature [20,24,25,26]. Identical Raman features were observed by a 633 nm CW laser excitation, thus confirmed the SERS effect of the sample. The laser power was set at 30 mW. After filtering and optical manipulation, about 1% of the power was incident on the sample. We found the strength of the SERS signal were quite consistent among samples with the same evaporation conditions. The signals were due to the trace amount of carbonaceous deposition on the sample surfaces during evaporation as reported in literature [20,26]. The carbon deposition on evaporated silver surface is common and often undesired because it might blur the signal and diminish the enhancement factor [27]. Because the purpose of the current study is to characterize the efficiency of the SERS substrates developed with the different techniques, we will take advantage of the carbonaceous deposition for the following analysis. We carefully investigated the composition of the sample surfaces deposited with various conditions by EDS (energy dispersive spectroscopy) and found the atomic percentage of carbon were all around 10%, but the difference in deposition duration might be several times this amount. This might be due to the sample and the camber having achieved an equilibrium during the evaporation. Typical values of EDS analysis are summarized in Table 1. Note that the carbon contents of each sample were on the same order. Because the Raman features from these carbon depositions were quite robust [20,28], we simply use the carbonaceous Raman signal for our following analysis. This could avoid many other influencing factors, such as uneven concentration of specific testing analyte, contaminations in solutions, or non-uniform distribution of the molecules after drying of the analyte solution. For example, we have tried to put the substrates in Rhodamine 6G solution and dried by blowing air. Probably due to the granular structure of the silver island film, the distribution of the molecules was not homogeneous. The signals varied in a wide range. However, for future sensing applications, it would be necessary to have specific molecules with stable affinity for signal reproducibility.

## 3. Results and Discussions

**Figure 5:**
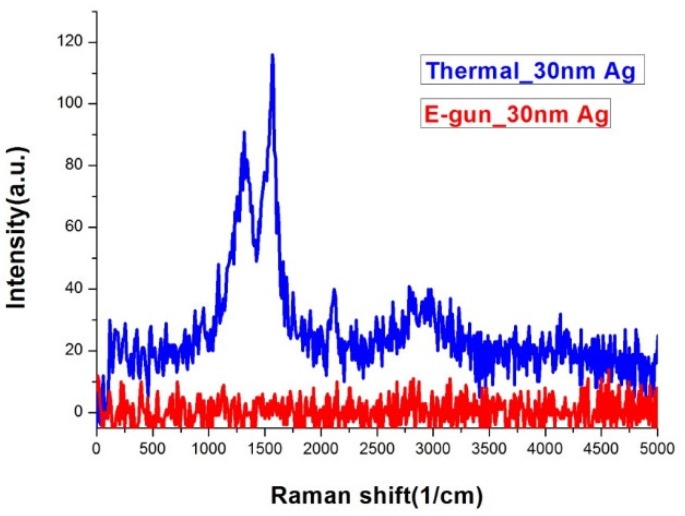
Raman spectra of silver films deposited by thermal and E-gun evaporation. The exposure time was 10 s.

Figure 5 shows the Raman spectra of silver island films deposited by thermal and E-gun evaporation under the same measurement conditions. Only the thermal one has obvious Raman signals. The EDS analysis confirms that both samples are composed of similar percentages of carbon as shown in Table 1. From the morphology and EDS analysis, we conclude that only silver films with island gaps have SERS signals. The gaps between the silver islands on the thermal evaporated sample are about 5–35 nm, which may induce localized surface plasmon resonance (LSPR). The Raman signals were boosted due to surface enhanced electric field [29,30].

**Figure 6:**
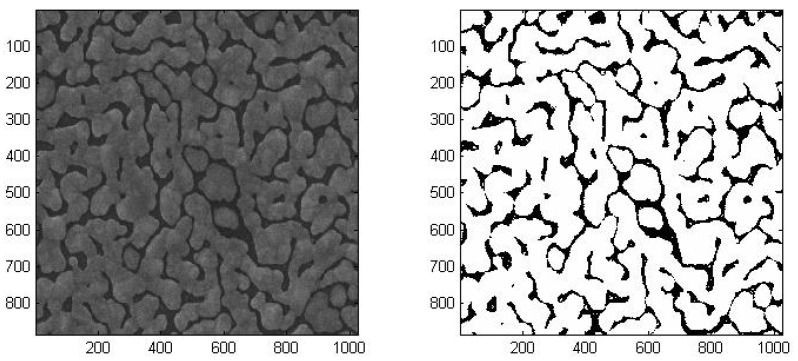
Schematic diagram of SEM image reconstruction. The figure on the left is the original SEM image and the figure on the right is the reconstructed binary image. The numbers on the axes are in nanometer.

In order to quantify the gap/silver ratio of each sample, we developed a pixel counting method to process the SEM images using Matlab. Firstly, we transformed the image to gray-scale. After analyzing the pixel intensities of the image, we could define a threshold value to distinguish pixels with or without silver. By comparing the pixels above and below the threshold, ratio of gap area over silver covered area can be determined. A comparison of the original SEM image and the image generated by the binary reconstruction method is shown in Figure 6. The gaps and the silver covered areas can be clearly identified. For each sample, we took four SEM images at four different points. The images were analyzed to get the ratios and averaged gap widths. The average densities and gap widths were shown in Table 2. The deviations of the densities between images from the same samples were within ± 3%.

**Figure 7:**
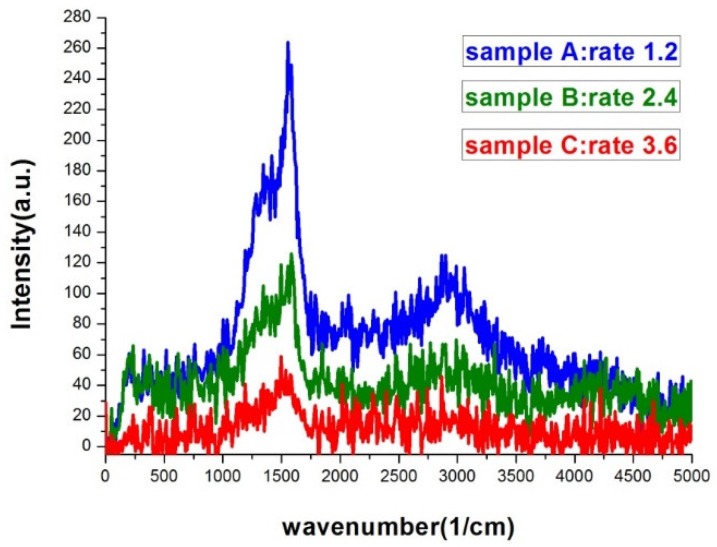
Raman spectra of silver island films deposited with evaporation rates of 1.2, 2.4, and 3.6 Å/s. The exposure time was 20 s.

**Figure 8:**
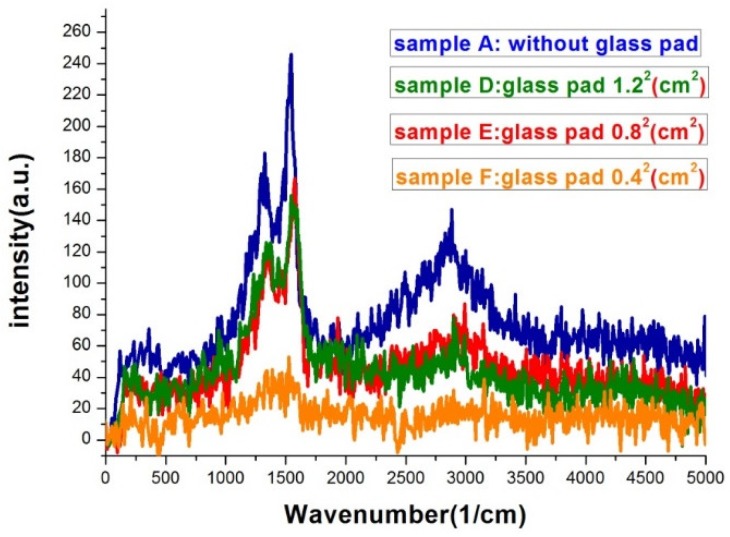
Raman spectra of silver island films with glass pads underneath during thermal evaporation. The evaporation rate was the same, 1.2 Å/s, for the four samples. The exposure time was 20 s.

The two key properties of film morphology to affect SERS intensity were the gap density and the width of the gap. Figure 7 was the Raman spectra of sample A, B, and C. The morphology difference between the three samples can be clearly seen in Figure 1. The higher the deposition rate, the wider the islands and the gaps would be. Sample A had the most compact island features. As seen in Figure 7, the SERS signal of sample A was at least 6-fold higher than that of sample C. Sample A, B, and C had similar gap ratios, however the average gap width of sample A was only about half of that of sample C. The rapid increase in signal with the shrinking of the gap width was the manifestation of LSPR induced SERS effect. Figure 8 was the Raman spectra of sample D, E, F, and one without bending. All samples in Figure 8 were deposited under the same thermal evaporation rate of 1.2 Å/s. Although the average gap widths of samples evaporated with bent substrates were smaller, the SERS signals were lower than the one without bending (condition A). The result conflict with what we expected. We will give our speculation about the unexpected result in the following discussions. In a closer examination of the signals from sample D and E, we found they are almost the same. The morphology difference between sample D and E was revealed in the SEM images of Figure 3. Sample D had a higher gap density, while the gap widths were smaller in sample E. This evidently showed that both gap density ratio and gap width played important roles in affecting the SERS efficiency. A smaller gap width indeed increased the signal. It is well-known that a smaller gap in nano-sized structure has a larger enhancement factor in SERS [8,9,31]. Therefore, although sample D had a larger gap density, the larger gap widths compromise the enhancement effect. Thus we did not see a significant difference in the SERS signals between the two samples.

When comparing the Raman signals from sample E and F, the signal intensity from the former is 3–4 folds larger than the latter, while the gap density of the former is only about twice as large as the latter and the gap width difference is less than 15%. The most likely reason for the smaller signal from sample F might be the gaps on the sample are vanished during substrate flattening as manifested in the SEM image and the carbon atoms are buried under the squeezed silver islands. Therefore, the excited carbon atoms and the collected signals are reduced.

**Figure 9:**
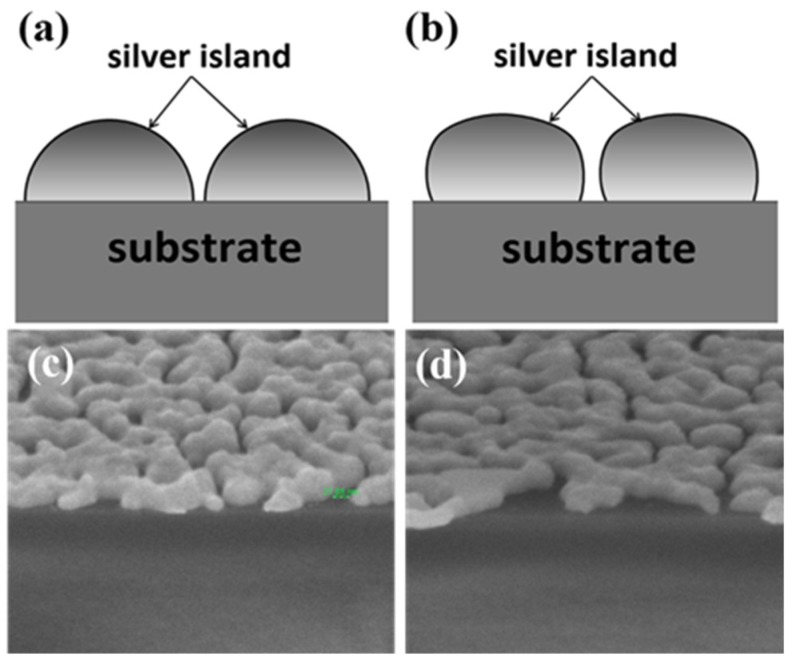
Schematic illustration of the boundary contour shapes of the islands developed by (**a**) typical thermal evaporation, (**b**) thermal evaporation with substrate bending. (**c**,**d**) are SEM images of 65° cross section of sample A and sample D with 4 × 10^5^ magnification, respectively.

After a carefully analysis of the data, we find other factors may also influence the enhancement effect which might be the reason of the unexpected results. Sample A and D had similar gap densities but the gaps on sample D were narrower. However, the Raman signals from sample D is not higher than that from sample A. Here we propose three possible reasons. One is the coupling efficiency difference due to the dissimilarity in geometry shape of the silver islands developed by the two methods. The excitation of surface plasmon is due to the scattering from topological defects on the surface, which are the sub-wavelength gaps. If we consider the boundary contours of the cross-sections of the islands developed by the two methods, the contour of the island deposited without glass pad underneath is relatively closer to a semicircle as schematically shown in Figure 9. While the island developed with substrate bending does not have such a smooth circular contour. The semicircular contour may have a better coupling efficiency for plasmons. The cross-section SEM images of the samples also reveal the difference in three-dimensional shape of the nano-islands developed using the different methods. Figure 9c is the cross-section SEM image of sample A. The shape of islands is closer to a semicircle with a smaller contact angle with the substrate. However, the shape is more oblate for sample D and the contact angle is larger as seen in Figure 9d. Secondly, the change in nano-island shape and film morphology would induce shift in resonance frequency, which might result in reduced signal. The third possible reason is that the buried mechanical strain on the silver island film due to substrate flattening might mitigate the SERS effect. There are some recent studies using strain or deformation of nanoparticle to alter the LSPR strength [23,32]. This is an interesting topic of research, and may require further investigation.

As seen above, substrate bending is an efficient way to control gap widths of the silver island film. For samples prepared with higher evaporation rates, due to the coarser geometry features, we have lower SERS enhancement as seen in Figure 7. The proposed substrate bending method can be used to reduce the gap size of the samples when using higher evaporation rate. Table 3 summarized the results when using different bending curvatures to reduce the widths of the gaps on samples evaporated with a rate of 3.6 Å/s (condition C). For conciseness, we took the Raman features at 1340 and 1580 cm^−1^ for comparison. The values were averaged from measurements of at least four points on the same sample. For samples without bending, the SERS intensity of sample C was less than one-fourth of that of sample A. When using substrate bending to reduce the gap size, the SERS intensity is largely increased. For the one bent by a 0.4^2^ cm^2^ glass pad underneath, the signal strength was almost as high as sample A. Because of the higher evaporation rate, the preparation time was greatly shortened. The reason for the largely enhanced SERS signal was mostly due to the reduced gap widths. Unlike in the case of sample F, because the samples prepared with higher evaporation rate had originally larger gap widths, the gaps would not vanish after substrate flattening and the carbon atoms in the gaps could still contribute significantly to the SERS signal. When using a 1.2^2^ cm^2^ glass pad to bend the substrate, the average gap widths reduced to 25.4 nm. However, there was a large variation on the width distribution from about 15 to 50 nm. For samples bent by 0.4^2^ cm^2^, and 0.8^2^ cm^2^ glass pads, the average gap widths are 15.5 and 17.3 nm, respectively. Some of the gap sizes were below 10 nm. The largely enhanced signals might be due to carbon atoms embedded in these reduced gaps.

**Figure 10:**
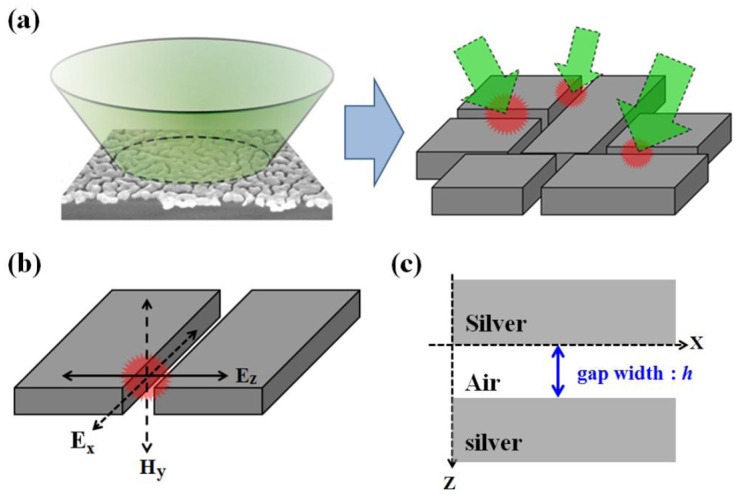
Schematic illustration of the structure for simulation. (**a**) Laser excitation and idealized silver islands as metal–insulator–metal (MIM) nanostructure. (**b**) Coordinate definition in the simulation. (**c**) Schematic diagram of the MIM structure in 2D.

**Figure 11:**
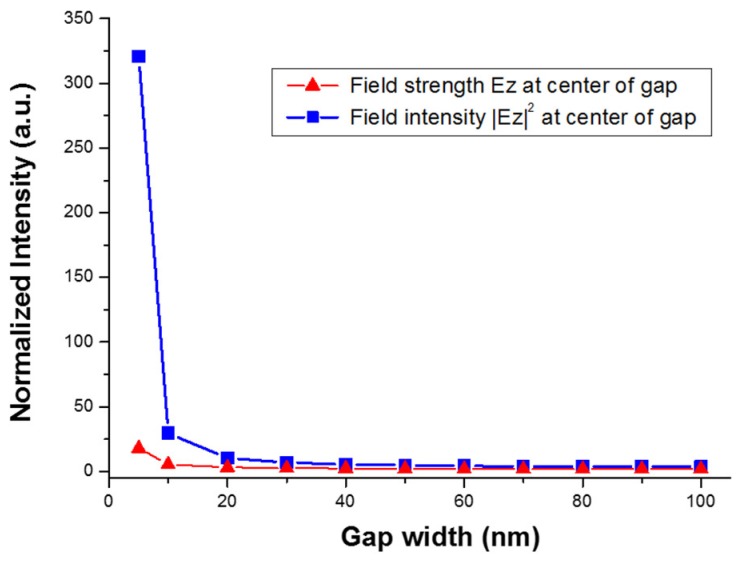
Simulation results of electrical field strength and intensity at center of the gap. The blue squares are the field intensity, and the red triangles are the field strength. The field strengths are normalized to the incident light.

In order to understand the enhancement due to gap width and ratio change, we used a metal–insulator–metal (MIM) nano-structure model to simulate the electrical field on the silver island film. Because of the irregular shape of the silver islands, it is difficult to quantify the geometry of the structure. There were very limited simulation works specifically for silver island films. The schematic for our simulation was illustrated in Figure 10. We used metal bars with a height of 30 nm to simulate the silver islands. The width of the metal bar was determined by the analyzed SEM image. By varying the size of the gap in between, we investigated the field strength at the center of the gap. The calculated TM electrical field (Ez) strength and intensity is shown in Figure 11. A clear trend of exponential increase in the field strength as the gap size reduces is seen in the figure. At a gap size of 10 nm, the field intensity is enhanced about 30-fold, which corresponds to a SERS enhancement of about 103. However, with a gap size smaller than 10 nm, the field intensity can be enhanced more than 300-fold, which would lead to a SERS enhancement factor of 105. Therefore, we can expect greatly enhanced SERS signal from these reduced nanogaps. The trend of enhancement was in accordance with what we have observed in the experiment.

Using the proposed method, we can efficiently control the gap size and density of the silver island film. The resulted enhancement was quite consistent among samples prepared with the same condition. This method allow us to generate large-area SERS-active substrate of high enhancement in a shorter time, which is desired for mass production. This kind of approach is readily applicable in flexible substrates for SERS or fluorescence enhancement applications [33].

## 4. Conclusions

In this paper, we confirmed that silver island films deposited by thermal evaporation have SERS effect as compared to the flat film developed by E-gun. We proposed a simplified method to control the gap density and morphology of the island film by bending the substrate while using a fixed evaporation rate. By comparing the Raman intensities of the island films produced by the different methods, we conclude that smaller gaps get better enhancement and larger gap–silver ratio also gives more signals. There is a trade-off between the two parameters. Besides, the morphology of the islands also influences the strength of SERS signal. For islands with smooth semicircular contours, the enhancement is more significant. Finally, the location of the analytes also plays an important role in the signal enhancement. The analytes should be placed in the gaps but not on the silver for optimal enhancement. Therefore, the best island film for SERS may be the one with high gap density and small gap size, the geometry of the island is close to semicircle in cross section and smooth. This new method provide us additional control on the morphology of the island film. We can thus generate small gap size SERS-active substrates with a higher evaporation rate in a shorter period. The SERS efficiency of substrate generated by the proposed method is comparable as the traditional method. This facile and cost-effective method is suitable for mass production of reproducible large-area SERS substrate for routine applications, such as environmental pollution monitoring and immunosensors. 

## Figures and Tables

**Table 1:** EDS analysis of 30 nm thick silver films deposited by thermal and E-gun evaporation.

Thermal Evaporation			E-Gun Evaporation		
Element	Weight %	Atomic %	Element	Weight %	Atomic %
C	5.69	10.57	C	3.04	8.35
O	38.94	54.27	O	23.19	47.74
Si	38.36	30.46	Si	24.66	28.92
Zn	8.82	3.01	Ag	49.1	15.00
Ag	8.18	1.69			
Totals	100.00		Totals	100.00	

**Table 2:** The gap densities and gap widths of samples developed with different conditions. The data shown are the average values of several SEM images taken from each sample.

Sample	Gap Density	Gap Width (nm)
A. rate 1.2 Å/s	15.93%	17.3 ± 5
B. rate 2.4 Å/s	17.05%	23.4 ± 7
C. rate 3.6 Å/s	17.40%	30.1 ± 8
D. glass pad 1.2^2^ (cm^2^) @ rate 1.2 Å/s	15.82%	15.7 ± 5
E. glass pad 0.8^2^ (cm^2^) @ rate 1.2 Å/s	13.18%	13.5 ± 5
F. glass pad 0.4^2^ (cm^2^) @ rate 1.2 Å/s	7.40%	11.6 ± 4

**Table 3:** The average Raman intensities at the 1340 and 1580 cm^−1^ features of samples prepared with various conditions.*

Raman Shift	Without Bending			With Substrate Bending		
	sample A rate 1.2 Å/s	sample B rate 2.4 Å/s	sample C rate 3.6 Å/s	glass 1.2^2^ (cm^2^) rate 3.6 Å/s	glass 0.8^2^ (cm^2^) rate 3.6 Å/s	glass 0.4^2^ (cm^2^) rate 3.6 Å/s
1340 cm^−1^	184.3	90.5	42.6	58.8	153.1	180.3
1580 cm^−1^	260.2	122.4	59.7	86.6	214.3	250.6

* The values were averaged from at least four points on the same sample.
